# An Evaluation of Portuguese Societal Opinion towards the Practice of Bullfighting

**DOI:** 10.3390/ani10112065

**Published:** 2020-11-07

**Authors:** Francisco Javier Diéguez, Yara Zau, Inés Viegas, Sara Fragoso, Patricia V. Turner, Gonçalo da Graça-Pereira

**Affiliations:** 1 Departamento de Anatomía, Producción Animal y Ciencias Clínicas Veterinarias, Facultad de Veterinaria, Universidad de Santiago de Compostela, Campus Universitario s/n, 27002 Lugo, Spain; 2 MYPZ—Farma&Vet, Av. Joaquim Agostinho 8; Loja B, Santa Cruz, 2560-065 A-Dos-Cunhados—Torres Vedras, Portugal; yarapz@gmail.com; 3 ICAAM–Mediterranean Institute of Agronomical and Environmental Sciences, Institute for Advanced Research and Formation, Évora University, Pólo da Mitra, Ap. 94, 7006-554 Évora, Portugal; inesviegas@gmail.com; 4 Centro Para o Conhecimento Animal. Av. Bombeiros Voluntários de Algés 40A, 1495-143 Algés, Portugal; bio.fragoso@gmail.com (S.F.); ggp.vet@gmail.com (G.d.G.-P.); 5 LabCAP—Instituto Superior de Estudos Interculturais e Transdisciplinares (ISEIT), Instituto Piaget de Almada, Avenida Jorge Peixinho, 30 Quinta da Arreinela, 2805-059 Almada, Portugal; 6 Department of Pathobiology, University of Guelph, Guelph, ON N1G 2W1, Canada; patricia.turner@crl.com; 7 Charles River Laboratories, Wilmington, MA 01887, USA; 8 Escola Superior Agraria de Elvas, Instituto Politécnico de Portalegre, Av. 14 de Janeiro 13, 7350-092 Elvas, Portugal

**Keywords:** animal ethics, animal welfare, demography, multiple correspondence analysis, Portugal, tauromachy

## Abstract

**Simple Summary:**

Bullfighting is one of the most controversial topics in animal welfare and ethics in recent years. This activity is an issue at the forefront of many animal welfare organizations. In the present study, an online questionnaire was used to seek Portuguese citizens’ opinions towards bullfighting and to relate these opinions to certain demographic characteristics. The majority of respondents had negative opinions about bullfights. Most questioned the artistic reasons to take the bull’s life in the name of culture and did not attend bullfighting events. However, the population interviewed was not representative of the Portuguese population. Men, older people, Roman Catholics, and people from rural areas (underrepresented in the study sample) showed a more favorable attitude towards bullfighting. Contrast between regions was also reflected; the districts where the most favorable opinions were collected (Satarém, Évora, Beja, and Portalegre) were those with the greatest presence of bull breeders. Public opinion research is an important policy-making instrument that could be useful in the face of possible initiatives to ban bullfighting at regional or country levels.

**Abstract:**

Bullfighting is a controversial sport that continues to be legally permitted in a number of countries around the world, including Portugal. The spectacle has attracted significant attention from animal protectionist groups for many years because of concerns for animal distress, pain, and suffering during the fights. While there has been strong support for the sport in Portugal in the past, there is a need to study social profiles regarding the acceptability of this sport before a case can be made for changes in regional and national legislation. In this study, Portuguese attendance patterns at bullfights were assessed in addition to public opinions on welfare and ethical aspects of bullfighting, based on demographic variables. Study participants (n = 8248) were largely recruited through Portuguese social media channels (respondents may not be representative of the Portuguese population). Questionnaire data were evaluated by means of frequency tables, multiple correspondence analyses, and a two-step cluster analysis. Most respondents had a negative opinion about bullfighting and perceived that bullfighting had no positive impact on the country. However, while most respondents thought that the bull suffered during bullfighting, the opinion regarding banning bullfighting was far from unanimous. Based on the demographic analysis, the profile of individuals with more favorable responses towards bullfighting were men > 65 years old, of Roman Catholic faith, of low- or high-income levels, from more rural areas of Portugal. Somewhat surprisingly, there was a tendency to favor bullfighting amongst veterinary professionals. We conclude that there were still large pockets of individuals who desire to maintain the practice of traditional bullfighting within Portuguese society, despite recognition of animal suffering during the event.

## 1. Introduction

Bullfighting, tauromachia or tauromachy, as it is frequently called, is a traditional exhibition seen in Spain, Portugal, and Southern France. It was introduced by Spaniards to Colombia, Ecuador, Venezuela, Peru, and Mexico dating back to the 16th century [1]. Portuguese-style bullfights are called *touradas* or *corridas de touros*, and each year, approximately 2500 bulls are used in fights in Portugal [2]. The Portuguese bullfight is conducted by a *cavaliero* (rider) on horseback, who stabs the bull with several *bandeiras* (small javelins). The bull is also challenged by a group of men on foot called *forcados*, who are usually unarmed and who work to subdue the bull [3]. 

In Portugal, unlike other countries, the bull is not killed in full view of the public. However, on most occasions, the bull is sent to a slaughterhouse after the fight, where it is slaughtered according to Portuguese regulations. The exception to this is in Barrancos, a town in Southern Portugal, in which a special legal dispensation has been granted to kill the bull during fights as part of a long-standing tradition.

Bullfighting across Portugal is not consistently seen across the country and most fights take place during summer [2]. Of the country’s 308 council areas, only 44 or approximately 15% have bullfighting activity, with Lisboa and Albufeira (in the Algarve) providing the main venues. Both the number of *touradas* and the number of spectators has generally declined in recent years. However, between 2016 and 2017, there was a 4.4% increase in bull fights, something that had not happened since 2010 [2]. 

Supporters of bullfighting consider it to be a deeply ingrained and integral part of the national culture and identity [4]. In addition, although poorly documented, the economic value of bullfighting in Portugal is thought to be significant in certain regions. Minor economic gains are realized at cattle ranches through breeding, raising, and caring for the bulls, with greater economic gains going to those working as entrepreneurs, bullfighters and their assistants, and arena staff [5]. Supporters also suggest that bullfighting attracts tourists; however, recent surveys suggest that the number of tourists attracted to this kind of activity is small. In fact, bullfighting might even be perceived as an unattractive event to tourists [6].

Due to the perceived suffering and distress of bulls during the fights there has been significant interest by animal protectionist groups to abolish bullfighting. Portuguese groups have been active in campaigning against bullfighting, and from 2002 this was their main campaign activity [7]. The Portuguese government has recently rejected a bill to ban bullfights that was submitted by PAN, the People-Animals-Nature party [8]. The bill was rejected by all major parties with few abstentions. Within the E.U., lawmakers in the European Parliament voted to approve an amendment to the 2016 EU budget indicating that EU subsidies should not go to farms that raise bulls for use in bullfighting. They added that such funding “is a clear violation of the European Convention for the Protection of Animals Kept for Farming Purposes” [9]. 

Some researchers have suggested that fighting bulls secrete large quantities of endorphins during the fight that help to mitigate pain [10,11]. Endorphins are hormones that can modulate physiologic responses to pain, but also to aversive stimuli [12]. Despite this, it seems evident that during wounding and other physical attacks that occur during bullfights, bulls exhibit behaviors indicative of distress including tail swishing, labored breathing, exhaustion, and reluctance to move [13]. A previous study also described severe anatomical damage to bulls after fights, concluding that this type of show clearly violates the minimum animal welfare standards and represents a clear expression of animal abuse [14]. In Portugal, the law 92/95 states that all unjustified violence against animals is forbidden, examples including acts that consist of unnecessarily inflicting death, cruel and prolonged suffering, or severe lesions to an animal [15]. Although animal abuse has been a part of tradition and culture, in the course of recent decades a number of practices have been questioned and many have been forbidden by law. Despite this, there is still legal protection of bullfighting in several countries on the grounds of preserving bullfighting as a national tradition [16]. In line with this, the Portuguese legal system criminalizes violence towards animals, but exceptions to this are granted for bullfights (and other entertainment using bulls) [7]. Since bulls are animals with the capacity to suffer pain, the reasons to oppose bullfighting would be the same as those to oppose other animal blood sports or practices that cause suffering and death of animals [17]. Pressures from the European Parliament exist to abolish bullfighting in those European countries where it still exists due to the duality of this activity occurring in the EU, in which animal welfare has been declared a priority [16]. In the described context, bullfighting goes against an animal’s rights and could be only permitted through a legal loophole expressly exempting bullfights from the laws of animal protection.

Although there seems to be a heightened sense of public contempt in many countries toward the treatment of animals and toward the use of animals in ‘sport’, several blood sports with animals still maintain a certain popularity in different areas [18]. In the context of understanding why people are attracted to blood sports and why they still exist, one significant reason includes a lack of understanding of the basic needs and well-being of animals [19]. This lack of understanding could lead to a lack of empathy though objectification. This could be attributed to a lack of education regarding basic animal care, behavior, and welfare [19]. In the case of bullfighting, a previous paper that evaluated the opinions of supporters included a primary motivation of having grown up in family environments related to bullfighting, the aesthetics of the show, that is, considering it as an artistic expression in which the bullfighter is trained in a certain style and elicits emotion through the act of the fight or even ecological reasons in that the existence of bullfighting preserves the breed of cattle and the typical ecosystem in which it is raised [20]. 

A study conducted well over a decade ago suggested that many Portuguese citizens believe that bullfights should be abolished due to their cruel and violent nature [21]. In that study, 51% of respondents indicated support for laws banning bullfighting, whereas 40% were opposed to changing the status quo [21]. However, recent informal polls have suggested that social division is still present on this topic in Portugal and there is a need to examine this issue more formally.

The aim of this study was to characterize Portuguese opinions regarding bullfighting by demographics variables, to better understand Portuguese societal support for updated animal welfare practices. 

## 2. Materials and Methods

### 2.1. Data Collection

Using the form function in Google Docs, a Portuguese language questionnaire, consisting exclusively of closed questions, was generated through consideration of existing literature to collect information regarding attitudes to bullfighting in Portugal [21,22,23,24]. Questions explored whether the respondents attended or had attended bullfighting shows. For those respondents that still attended bullfighting, questions inquired about their motivation to attend, the age they started to attend these events, and whether they would continue to attend if the bull was replaced by another animal (i.e., a dog, that is, a domestic animal towards which people generally show higher level of empathy) or a robot (substitution by something that does not imply animal suffering). For those who had attended at some point but no longer did, questions asked about their reasons for discontinuing. The core part of the questionnaire explored general opinions regarding bullfighting: whether the respondent considered that bullfighting was beneficial for the economy, tourism or culture of Portugal, whether bullfighting and related supporting activities should receive public funding, if they thought that bullfighting generates positive connotations for the country, whether it has greater, lesser, or equal artistic value than painting, and respondents’ opinions on the bull’s capacity to suffer pain compared to a dolphin, dog, or human. The survey also sought an opinion as to whether the bull suffers during fights and if respondents thought that the fighting bull breed would disappear if bullfighting did not exist. Finally, respondents were asked if bullfighting should be allowed to continue. Demographic characteristics including gender, age, occupation, education level, monthly income, religion, region of residence, habitat (rural or urban), and whether the respondent had a relative linked to the bullfighting industry were also collected. Descriptive statistics regarding the demographic characteristics of the studied population and the general population of Portugal are shown in Table 1.

From December 2016 to March 2017. The online survey was communicated through the social media such as Facebook, Instagram and LinkedIn. Moreover, via e-mails, it was shared to personal contacts of the research group members having been chain-shared by multiple users. Before starting the questionnaire, the online survey, included a brief description of the study and its aim. All questionnaire information collected was anonymous and participation was voluntary. No incentives were provided for participating in this study. Prior to dissemination, the questionnaire was first administered to 10 people to ensure clarity of questions. Minor edits were incorporated before widespread administration to the general public. In all, 8248 responses were obtained (Table 1) and all Portuguese districts were represented in responses (Table 2).

### 2.2. Statistical Analysis

All statistical tests were conducted using SPSS 15.0 (SPSS Inc., Chicago, IL, USA). To evaluate raw data, frequency tables were generated for each question. Following this, a multiple correspondence analysis (MCA) was performed [25]. The goal of the MCA is to reduce a set of possibly correlated variables (including bullfighting attendance patterns, demographic variables, and opinions) to a smaller group of linearly noncorrelated ones (dimensions). In this study, the number of dimensions was set to two to allow for a two-dimensional graphical representation. The position of the full set of categories for each investigated variable (category-points) in the MCA graph is the basis for revealing relationships among them: variable categories with a similar profile tend to be grouped together whereas those negatively correlated are positioned on opposite sides of the graph. The origin of the graph reflects the weighted average of the categories for each variable considered in the study (centroid of each variables). As a result, the closer a category point is to the origin, the closer it is to the average profile. From the MCA, the correlation matrix of the resulting variables (once optimal scaling had been performed) was also completed in the analysis. Finally, a two-step cluster analysis (TSCA) was performed to identify clusters of people with a similar opinion about bullfighting.

## 3. Results 

In terms of respondent demographics, approximately 61% were female (vs. 53% in the Portuguese general population) and 84% were less than 48 years old (whereas almost 53% were less than 45 years old in the general population) (Table 1). Most respondents were employed full-time with >95% indicating that they did not work in the veterinary profession. Just under 70% of respondents had undergone some post-secondary education (~25% in Portugal) and 40% had a net monthly income < 1590 euros (vs. 45% in Portugal). Approximately half of respondents identified themselves as Roman Catholics (56% in Portugal) and ~75% lived in an urban environment (vs. ~64% in Portugal). To summarize, most respondents were relatively young, well-educated, and urban-dwelling women. 

Approximately 50% of respondents never attended bullfighting events, while almost 20% had attended at some point but no longer did primarily because of animal welfare concerns, whereas the rest of the participants continued to attend bullfighting events. Of these, most began to attend bullfighting before the age of 18 and they attended for cultural reasons. Similarly, most indicated that they would stop attending if the bull was replaced by another animal (i.e., a dog) or a robot (Table 3). 

According to the results from this survey, most respondents had a negative opinion about bullfighting with the predominant perception being that bullfighting has no positive impact on Portuguese culture or tourism. With respect to its impact on economy, there was more discrepancy in responses, but again, the majority felt that this activity should not receive public funding. In general, it was believed among those surveyed that bullfighting does not generate positive press for the country, and in line with this, bullfighting was given less artistic value than painting (Table 4). It was widely accepted that the bull suffers during bullfights and, in line with this, most respondents indicated that a bull’s capacity to suffer pain is like that of other animals or humans. Additionally, a greater number of respondents (although they were still minority) believed that the fighting bull would disappear as a breed if bullfighting did not exist. Only 30% of respondents considered that bullfighting should be allowed to continue (Table 4).

**Figure 1:**
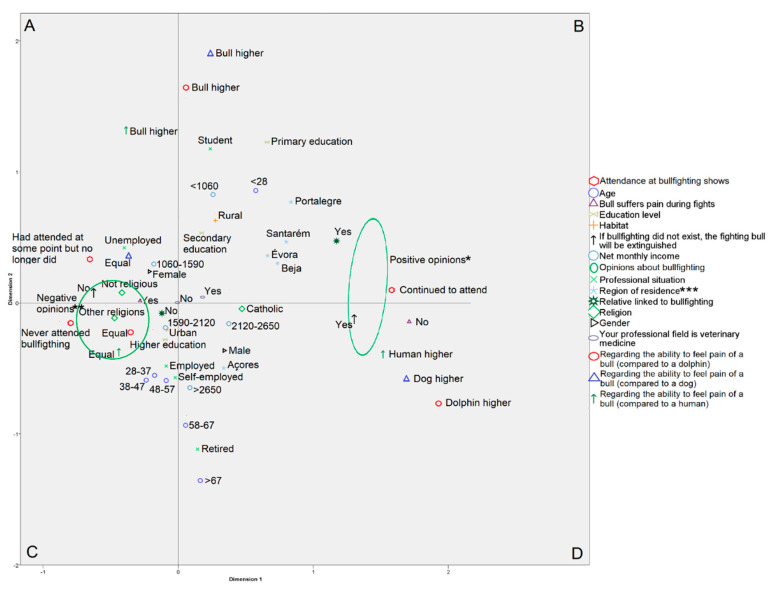
Multiple correspondence analysis for the different demographic and opinion variables. Variables with a similar profile tend to be grouped together whereas those negatively correlated are positioned on diagonally opposite sides of the graph. The origin of the graph reflects the weighted average for each demographic or opinion variable considered. The closer a variable is to the origin, the closer it is to the average profile of the survey respondents. * Positive opinions: location of the category points “Yes” for the questions “Bullfighting favors economy”, “Bullfighting favors tourism”, “Bullfighting favors economy”, “Bullfighting must receive public founds”, “Bullfighting generates positive connotations for the country” and “Bullfighting continuity should be allowed” and the category points “Bullfighting higher” and “Equal” for the question “Bullfighting has greater, less or equal artistic value than painting”. ** Negative opinions: location of the category points “No” and “Painting higher” for the same questions. *** Only the districts with opinions more favorable to bullfighting are shown. The rest are located in or near quadrants A and C.

The MCA, in which the data have been standardized, explained 30% of the variance of the data on demographic and bullfighting opinions from 8248 respondents. The percentage of variance explained by the first dimension was ~20%, and for the second dimension was 11.3%. The main results of the MCA are presented in Figure 1. Dimension one clearly differentiates between people with positive and negative opinions regarding bullfighting. The correlation matrix of the transformed variables considered in the study (after optimal scaling) is presented in Appendix A (Table A1).

According to the TSCA, two clusters were formed. Cluster 1, which includes 73% of respondents, was the group with unfavorable opinions towards bullfighting. These individuals mostly did not attend or had stopped attending bullfighting. Cluster 2, representing 27% of respondents, was the group with a more favorable view towards bullfighting, who mostly still attended events and were least likely to recognize the suffering of the animal. Table 5 and Table 6 representing within-cluster percentages demonstrate how each opinion or demographic variable is split within each cluster.

Unfavorable views of bullfighting were expressed more commonly by women, amongst those with average income levels, those living in urban areas, and in individuals with higher education levels. The categories corresponding to men, high- or low-income levels, rural living, and lower education level lay somewhere in between (Figure 1), indicating that among these different categories, opinion was more divided. Older, retired individuals were noted to value bullfighting positively more often, and to a lesser extent those less than 28 years of age (although the categories corresponding to rural habitat, low income level, and age under 28 were close in the MCA graph and, are co-correlated) (Figure 1). Results from the TSCA confirm these demographic patterns. For example, 41.1% of men but only 18.6% of women were in cluster 2. Regarding age, the category corresponding to those >67 years old was the one with the highest percentage in cluster 2 (Table 6). Individuals who identified themselves as non-practicing or agnostic as well as people indicating that they subscribed to a religion other than Roman Catholicism tended to have more negative opinions about bullfighting. The category corresponding to Catholics was located somewhere between positive and negative opinions (Figure 1). Thus, the TSCA indicated that 91.9% of those who declared themselves agnostic were in cluster 1, while only 53.3% of Roman Catholics were in this cluster (Table 6). Interestingly, respondents who indicated that their profession was veterinary medicine had a slightly more favorable opinion towards bullfighting than those who did not (Figure 1). Specifically, the percentage of veterinarians in cluster 2 was 33.0% compared to 26.8% in the respondents whose profession was not veterinary medicine (Table 6).

Regarding the place of residence, the MCA graph indicated that favorable responses to bullfighting occurred in individuals living closer to the districts of Satarém, Évora, Beja, and Portalegre (i.e., bordering districts that extend from the center to the south of Portugal) and to a lesser extent in Açores (Figure 1). People from northern districts, in addition to Faro, tended to have the most unfavorable opinion towards bullfighting. The TSCA indicated that the districts of Satarém, Évora, Beja, and Portalegre were the only ones in which the percentage of respondents that fit in cluster 2 exceeded 50%, while in Açores they were approximately 41.1% (data not shown). 

## 4. Discussion

The results of this survey about Portuguese societal attitudes towards bullfighting indicated that the majority of those responding held negative opinions about the sport. Although bullfighting is still popular with thousands of fans across Portugal, it has lost its relevance in a more modern society. Interestingly, most respondents who had stopped attending bullfighting did so for animal welfare reasons, which indicated a growing social awareness towards this issue. Despite this, the popularity of bullfighting has extended beyond its traditional home ground (Portugal, Spain, and South and Central America) to reach new attendees in North America, Japan, and Eastern Europe [26]. 

Bullfighting fans claim that there are moral arguments in favor of the activity and that supporting it is a legitimate ethical option [27]. Likewise, supporters want to separate themselves from other animal blood sports fans by emphasizing their respect for animals and conservationism. They indicate that this is shown by the fact that bullfighting allows producers to preserve the cattle breed and that maintenance of bulls for bullfighting contributes to the maintenance of a traditional pasture ecosystem [17]. The cattle breed is considered unique for bullfighting fans since this breed has been traditionally selected for particular characteristics and behavioral traits (i.e., aggressiveness, strength, and mobility) [28]. Certainly, fans deeply appreciate the qualities that the bull embodies, but according to our results, they often do not recognize the suffering of the animal during the event. Others have suggested that spectators are fully aware of the pain and suffering inflicted on the bulls, but that the pain and suffering do not matter to them because of a callous or hedonistic viewpoint [29].

According to our results, most spectators indicated that they started to attend bullfights before the age of 18. This coincides with information from other studies suggesting that many bullfighting fans grew up in family environments in which there was a fondness for bullfighting [20]. To prevent the harmful effects that viewing bullfighting could have on children, the United Nations recommends that those overseeing bullfighting spectacles prohibit the participation of children under 18 years of age in bullfighter schools and as spectators in bullfighting events. Witnessing a bullfight could result in psychological trauma as well as a reduction in moral judgement and empathy. Others have argued that another possible consequence is that children could become accustomed to violence and become apathetic later when confronted with a violent incident [30]. This seems unlikely in that children who grow up in conditions favorable to bullfighting are simultaneously embedded within a rational and democratic society [27]. From the perspective of bullfighting schools, they claim to teach tauromachic as well as desirable virtues, such as effort, discipline, perseverance, humility, loyalty, and love for traditions. 

Our results concerning common social opinions are comparable to previous studies on this subject in Portugal. In a previous study in which 1064 people were interviewed by telephone, Monteiro et al. (2007) stratified responses according to origin and gender and determined that 51% of respondents were in favor of banning *touradas*, while 39% were opposed. The remaining 10% did not have a strong opinion one way or the other. In both studies, amongst men and people living in rural areas, the opinion regarding continuation of bullfighting tended to be more favorable. However, Monteiro et al. (2007) did not evaluate responses by age group [21]. In another study carried out in Spain, older men, those retired, and those of rural origin were identified as having the most favorable attitudes toward bullfighting [22]. Virtually all studies about animal activist group demographics have noted that women outnumber men among rank and file activists [31]. Research on the preponderance of women advocating for animal rights suggest that this is a result of women’s socialization. It emphasizes a relational orientation of care and nurturing that extends to animals’ and women’s experiences with structural oppression that might make them more disposed to egalitarian ideology, which creates concern for animal rights [32]. Moreover, more men than women support animal research, hunt animals for recreation, and engage in animal cruelty [33]. A previous study went further and stated that bullfighting is a male-focused ritual and masculine values frame the entire event [34].

From a different viewpoint, given rural individuals’ greater utilitarian attitudes toward animals, these people may view this activity as a function of costs and benefits, making it easier to justify the use of animals in entertainment, even if some animal suffering occurs [35]. In the present study, the responses from rural areas were more closely correlated to lower income levels, which could partly explain why in this income group positive attitudes appeared more frequently towards bullfighting, followed by the highest income groups. Previous studies indicated that younger age groups tend to show more concern for animals and animal welfare than older age groups. Additionally, older people showed higher levels of cultural conservatism, which encompasses the endorsement of traditional values [36]. The variable of age is also related to other variables, such as the professed religion or educational level, since young and middle-aged people more often tend to declare themselves non-practicing/agnostic and to have higher education levels [37]. Regarding religion, within the Iberian Peninsula, bullfighting still occurs at times within the scope of local or regional Catholic commemorations. Frequently before the fights, the bullfighter himself carries out a ritual closely linked to Catholic religious beliefs [38]. That is, after the ceremony of “dressing”, the bullfighters are placed in front of a chapel. This domestic altar is made up of numerous stamps, medals, images, etc., that bullfighters have acquired during their visits to various sanctuaries or that have been given to them by family, friends, and followers. The bullfighter, while standing in front of these objects, prays for success in the arena. It has been stated that more religious people demonstrated less positive (less humane) attitudes toward animal treatment than did more liberally religious (or less religious) individuals [39]. Religiosity has also become associated with a conservative orientation toward politics, primarily based on a cultural conservatism encompassing traditional stances [40,41]. 

Regarding income level, the highest levels of approval for bullfighting were observed in those respondents with either the lowest or highest income levels, while those with intermediate incomes least supported the activity. Lower incomes were primarily found in rural areas, while those with the highest incomes have also been associated with a greater level of economic and cultural conservatism [42].

Interestingly, the percentage of veterinarians in profile 2 (positive attitudes towards bullfighting) was higher than in the general population. It could be that amongst these individuals, responses were related to utilitarian arguments balancing the cost of entertainment for the public against suffering of relatively low numbers of animals and the generally good living conditions of these bulls versus the conditions for life and death for intensively-raised animals [43]. Additionally, many veterinarians may see bullfighting as an employment opportunity. Given the relevance of assuring that the bull is healthy and in perfect condition for the bullfight, veterinarians play an important role in the preparation and development of the show [44]. Despite these findings, there are anti-bullfighting activists in the veterinary sector (even leading associations against bullfighting), amongst veterinarians, and within veterinary faculties. Similarly, there were conflicting thoughts amongst the general population. While almost 85% of respondents indicated that they thought that the bull suffered during bullfighting, only 65% would ban bullfighting for animal cruelty reasons. Although most respondents indicated that they believed that bull’s capacity to suffer pain was equal to that of another animal or human, respondents corresponding to profile 2 considered that the bull’s capacity to suffer pain was less. It has been suggested that under conditions of extreme stress, production of endorphins and other metabolites may alleviate some part of perceived pain, but a reduction of pain would be replaced by marked distress or fear [31]. Even if one accepts that these bulls live better lives than other cattle raised for food production, this does not justify the distress and pain to which the bulls are subjected to during the bullfight. 

In Portugal, the largest number of bullfighting events are concentrated in the districts of Lisbon (the most populous city in Portugal and also the region with the most tourists) and Faro (another important tourist area) [2]. However, the districts in which the most favorable opinions were collected (Satarém, Évora, Beja, and Portalegre) are those with the greatest presence of bull breeders [45]. In these districts, the culture of bullfighting is probably more deeply rooted and because they are more rural, the population may tend to favor the preservation of primary economic activities. In Açores, and especially on the island of *Terceira*, there exists a particular type of bullfight (*touradas a corda)*. In this case, the bull is led along a designated course by means of a rope tied around its neck while the bull is taunted and teased by players (called *pastores*) who have no intent to kill the animal.

A possible limitation of this study is that people that have a vested interest in the topic were more inclined to complete the survey [46]. The population interviewed may not be representative of the Portuguese population. When the study was conducted, the percentage of men in Portugal was 47% (39% in the studied population). In addition, the percentage of people over 65 years of age was 21%, whereas amongst the studied population (including even those over 57 years of age) it was only 7%. People living in rural areas in Portugal represented 35.3% of the population (vs. 24.9% in the studied population) and people with only primary education 46.3% (vs. 1.5% in the studied population) [24]. Men, and especially older and rural dwellers, are least likely to be connected with social media [47]. This also leads us to infer that the public opinion regarding bullfighting in the general population of Portugal could be somewhat more divided than observed, since men, older individuals, and those living in rural areas had more positive opinions about bullfighting in our survey.

## 5. Conclusions

In summary, the profile of individuals with more favorable responses to bullfighting were men, >65 years old, of Roman Catholic faith, of low- or high-income levels, and from more rural areas. Amongst veterinary professionals there was also a tendency to favor bullfighting compared to the rest of the Portuguese population. Favorable opinions also occurred more often amongst those living in the districts of Satarém, Évora, Beja, and Portalegre, and to a lesser extent in Açores. Women, those identifying themselves as agnostic or non-Roman Catholic, individuals with an intermediate income level, and those from more urban areas evinced more negative opinions about bullfighting. Although suffering of the bull during the bullfighting event was generally recognized, there was still division over banning bullfighting within Portuguese society, and general initiatives to ban bullfighting have not found widespread favor by the Portuguese government or its citizens. 

## Figures and Tables

**Table 1:** Descriptive analysis of the survey respondents and the general population of Portugal (N = 8248 individuals).

Variable	Studied Population, Frequency (%)	Portugal, %
Gender		
Female	5035 (61.0%)	53%
Male	3213 (39.0%)	47%
Age		
<28	3535 (42.9%)	(<24) * 24.9%
28–37	1976 (24.0%)	(25–34) 12.7%
38–47	1434 (17.4%)	(35–44) 15.1%
48–57	766 (9.3%)	(45–54) 13.5%
58–67	404 (4.9%)	(55–65) 12.8%
>67	133 (1.6%)	(>65) 21.0%
Occupation		
Self-employed	1254 (15.2%)	11.8%
Employed	3769 (45.7%)	34.8%
Student	2353 (28.5%)	19.3%
Retired	341 (4.1%)	29.1%
Unemployed	531 (6.4%)	5.0%
Occupation other than veterinary medicine	7901 (95.8%)	>99.9%
Occupation veterinary medicine	345 (4.2%)	<0.1%
No response	2 (0.0%)	
Education		
Primary education	123 (1.5%)	46.3%
Secondary education	2380 (28.9%)	28.4%
Higher education	5741 (69.6%)	25.3%
No response	4 (0.0%)	
Net monthly income		
<1060 €	1566 (19.0%)	20.0%
1060–1590 €	1628 (19.7%)	25.7%
1590–2120 €	1727 (20.9%)	21.1%
2120–2650 €	1343 (16.3%)	18.1%
>2650 €	1627 (19.7%)	15.1%
No response	357 (4.3%)	
Religion		
Non-practicing/agnostic	3503 (42.5%)	40%
Catholic	4067 (49.3%)	56%
Other religions	678 (8.2%)	4%
Habitat		
Rural	2069 (25.1%)	35.3%
Urban	6179 (74.9%)	64.7%

* According to the age groups distribution provided by the Instituto Nacional de Estatística of Portugal.

**Table 2:** Self-declared frequency (percentage) of questionnaires obtained from each Portuguese district and distribution of the total Portuguese population in the different districts.

District	Frequency (%)	Distribution of Total Portuguese Population (5)
Açores	238 (2.9%)	2.5%
Aveiro	272 (3.3%)	6.9%
Beja	141 (1.7%)	1.4%
Braga	317 (3.8%)	8.5%
Bragança	27 (0.3%)	1.3%
Castelo Branco	82 (1.0)	1.9%
Coimbra	251 (3.0%)	4.3%
Évora	379 (4.6%)	1.6%
Faro	225 (2.7%)	4.4%
Guarda	60 (0.7%)	1.5%
Leiria	338 (4.1)	0.5%
Lisboa	2841 (34.4%)	22.6%
Madeira	75 (0.9%)	2.5%
Portalegre	162 (2.0%)	1.1%
Porto	865 (10.5%)	18.2%
Santarém	622 (7.5%)	4.5%
Setúbal	804 (9.7%)	8.5%
Viana do Castelo	118 (1.4%)	2.4%
Vila Real	60 (0.7%)	2.0%
Viseu	98 (1.2%)	3.6%
No response	273 (3.3)	

**Table 3:** Summary of respondents’ attendance at bullfighting events (N = 8248 individuals).

Attendance at bullfighting shows	**Response**	**Frequency (%)**	**Response**		**Frequency (%)**
	Never attended	4332 (52.5%)			
	Had attended but no longer do	1623 (19.7%)	Due to animal welfare		1227 (75.6%)
			Loss of interest		294 (18.1%)
			Stop liking it		102 (6.3%)
	Still attended	2293 (27.8%)	Starting age	<18	2103 (91.7%)
				18–25	133 (5.8%)
				>25	57 (2.5%)
			Reason to attend	Cultural	1970 (85.9%)
				Religious	73 (3.2%)
				Economic	106 (4.6%)
				No response	144 (6.3%)
			If the bull was replaced by another animal (i.e., dog), would you continue to attend?	Yes	20 (0.8%)
				No	2185 (95.3%)
				No response	88 (3.9%)
			If the bull was replaced by a robot, would you continue to attend?	Yes	92 (4.0%)
				No	1949 (85.0%)
				No response	252 (11.0%)

**Table 4:** Summary of respondents’ opinions regarding bullfighting (N = 8248 individuals).

Bullfighting….	Frequency (%)
Favors economy	
Yes	2931 (35.5%)
No	4619 (56.0%)
No response	698 (8.5%)
Favors tourism	
Yes	2685 (32.6%)
No	5137 (62.3%)
No response	426 (5.2%)
Favors culture	
Yes	2489 (30.2%)
No	5546 (67.2%)
No response	213 (2.6%)
Must receive public funds	
Yes	2006 (24.3%)
No	5888 (71.4%)
No response	354 (4.3%)
Generates positive connotations for the country	
Yes	1924 (23.3%)
No	5298 (64.2%)
Indifferent	1026 (12.5%)
Has greater, less or equal artistic value than painting	
Painting higher	5933 (71.9%)
Equal	1484 (18.0%)
Bullfighting higher	831 (10.1%)
Bull suffers during fights	
Yes	6985 (84.7%)
No	951 (11.5%)
No response	312 (3.8%)
Regarding the ability to feel pain of a bull (compared to a dolphin)	
Dolphin higher	1051 (12.7%)
Equal	6849 (83.0%)
Bull higher	348 (4.2%)
Regarding the ability to feel pain of a bull (compared to a dog)	
Dog higher	1193 (14.5%)
Equal	6857 (83.1%)
Bull higher	198 (2.4%)
Regarding the ability to feel pain of a bull (compared to a human)	
Human higher	1402 (17.0%)
Equal	6458 (78.3%)
Bull higher	388 (4.7%)
Relative linked to bullfighting	
Yes	1184 (14.4%)
No	7064 (85.6%)
Fighting bull breed would disappear if bullfighting did not exist	
Yes	2584 (31.3%)
No	4911 (59.5%)
No response	753 (9.1%)
Bullfighting continuity should be allowed or not	
Yes	2501 (30.3%)
No, mainly for animal welfare	5321 (64.5%)
No, for reasons other than animal welfare (such as negative effects on the culture or image of the country)	199 (2.4%)
No response	227 (2.8%)

**Table 5:** Composition of opinions regarding bullfighting in Portugal as obtained by a two-step cluster analysis.

Variable	Cluster 1	Cluster 2
Bullfighting favors economy		
Yes	18.9%	81.1%
No	99.2%	0.8%
Bullfighting favors tourism		
Yes	10.5%	89.5%
No	99.4%	0.6%
Bullfighting favors culture		
Yes	1.6%	98.4%
No	99.8%	0.2%
Bullfighting must receive public funds		
Yes	0.7%	99.3%
No	96.7%	3.3%
Generates positive connotations for the country		
Yes	2.4%	97.6%
No	99.0%	1.0%
Indifferent	41.8%	58.2%
Has greater, less or equal artistic value than painting		
Painting higher	95.6%	4.4%
Equal	14.4%	85.6%
Bullfighting higher	5.7%	94.3%
Bull suffers during fights		
Yes	84.1%	15.9%
No	0.3%	99.7%
Regarding the ability to feel pain of a bull (compared to a dolphin)		
Dolphin higher	0.3%	99.7%
Equal	83.9%	16.1%
Bull higher	44.5%	55.5%
Regarding the ability to feel pain of a bull (compared to a dog)		
Dog higher	8.0%	92.0%
Equal	84.4%	15.6%
Bull higher	40.3%	59.7%
Regarding the ability to feel pain of a bull (compared to a human)		
Human higher	8.9%	91.1%
Equal	85.2%	14.8%
Bull higher	79.4%	20.6%
Relative linked to bullfighting		
Yes	27.7%	72.3%
No	80.6%	19.4%
Fighting bull breed would disappear if bullfighting did not exist		
Yes	13.9%	86.1%
No	97.7%	2.3%
Bullfighting continuity should be allowed or not		
Yes	0.7%	99.3%
No, mainly for animal welfare	100.0%	0.0%
No, for reasons other than animal welfare	99.9%	0.1%

**Table 6:** Within cluster composition of demographic profiles in Portugal within the clusters obtained by a two-step cluster analysis.

Variable	Cluster 1	Cluster 2
Attendance to bullfighting shows		
Never attended	98.3%	1.7%
Attended but stopping do it	97.4%	2.6%%
Attend	0.6%	99.4%
Gender		
Female	81.3%	18.7%
Male	59.0%	41.0%
Age		
<28	65.7%	34.3%
28–37	77.7%	22.3%
38–47	81.3%	18.7%
48–57	75.8%	24.2%
58–67	71.2%	28.8%
>67	59.6%	40.4%
Occupation		
Self-employed	74.4%	25.6%
Employed	74.6%	25.4%
Student	65.9%	34.1%
Retired	70.5%	29.5%
Unemployed	87.6%	12.4%
		
Professional field is veterinary medicine	67.0%	33.0%
Professional field different to veterinary medicine	73.2%	26.8%
Education		
Primary education	48.0%	52.0%
Secondary education	67.5%	32.5%
Higher education	75.9%	24.1%
Net monthly income		
<1060 €	65.7%	34.3%
1060–1590 €	78.6%	21.4%
1590–2120 €	76.8%	23.3%
2120–2650 €	74.4%	25.6%
>2650 €	67.7%	32.3%
Religion		
Non-practicing/agnostic	91.9%	8.1%
Catholic	53.3%	46.7%
Other religions	88.5%	11.5%
Habitat		
Rural	64.5%	35.5%
Urban	75.9%	24.1%

## Appendix A

**Table A1:** Correlation matrix of the transformed variables (after optimal scaling) on demographic and bullfighting opinions in Portugal.

	Bull Suffers Pain during Fights	Fighting Bull Breed Would Disappear If Bullfighting Did not Exist	Regarding the Artistic Value (with Respect to Painting)	Bullfighting must Receive Public Funds	Bullfighting Favors Culture	Bullfighting Favors Economy	Bullfighting Favors Tourism	Bullfighting Generates Positive Connotations for the Country	Bullfighting Continuity Should Be Allowed or Not	Age	Sex	Religion	Relative Linked to Bullfighting	Habitat	Region of Residence	Education	Occupation	Professional Field Is Veterinary Medicine	Net Monthly Income	Suffering Capacity of Bulls (with Respect to Dolphins)	Suffering Capacity of Bulls (with Respect to a Dogs)	Suffering Capacity of Bulls (with Respect to Humans)	Attendance to Bullfighting Shows
Bull suffers pain during fights	1.000	0.486	0.497	0.535	0.530	0.444	0.480	0.529	0.537	0.088	0.145	0.224	0.267	0.044	0.167	0.060	0.079	0.017	0.061	0.485	0.488	0.493	0.518
Fighting bull breed would disappear if bullfighting did not exist	0.486	1.000	0.681	0.724	0.802	0.683	0.723	0.701	0.808	0.096	0.237	0.333	0.349	0.095	0.310	0.067	0.109	0.058	0.103	0.450	0.471	0.495	0.784
Regarding the artistic value (with respect to painting)	0.497	0.681	1.000	0.730	0.767	0.624	0.697	0.713	0.781	0.124	0.196	0.332	0.349	0.114	0.291	0.128	0.105	0.033	0.096	0.455	0.472	0.485	0.768
Bullfighting must receive public funds	0.535	0.724	0.730	1.000	0.823	0.677	0.737	0.757	0.840	0.120	0.206	0.355	0.385	0.103	0.316	0.097	0.104	0.021	0.091	0.473	0.493	0.515	0.813
Bullfighting favors culture	0.530	0.802	0.767	0.823	1.000	0.758	0.830	0.798	0.945	0.115	0.247	0.387	0.383	0.104	0.339	0.091	0.106	0.020	0.115	0.506	0.522	0.545	0.888
Bullfighting favors economy	0.444	0.683	0.624	0.677	0.758	1.000	0.773	0.675	0.767	0.117	0.231	0.314	0.303	0.098	0.285	0.052	0.122	0.030	0.074	0.425	0.447	0.462	0.736
Bullfighting favors tourism	0.480	0.723	0.697	0.737	0.830	0.773	1.000	0.757	0.841	0.134	0.229	0.363	0.359	0.109	0.313	0.100	0.124	0.024	0.113	0.456	0.484	0.504	0.806
Bullfighting generates positive connotations for the country	0.529	0.701	0.713	0.757	0.798	0.675	0.757	1.000	0.806	0.118	0.201	0.366	0.380	0.107	0.314	0.116	0.108	0.011	0.089	0.472	0.495	0.517	0.790
Bullfighting continuity should be allowed or not	0.537	0.808	0.781	0.840	0.945	0.767	0.841	0.806	1.000	0.118	0.247	0.395	0.386	0.113	0.341	0.095	0.108	0.026	0.113	0.508	0.529	0.554	0.912
Age	0.088	0.096	0.124	0.120	0.115	0.117	0.134	0.118	0.118	1.000	−0.115	0.038	0.146	0.132	0.080	0.107	0.521	0.039	0.096	0.146	0.163	0.156	0.140
Sex	0.145	0.237	0.196	0.206	0.247	0.231	0.229	0.201	0.247	−0.115	1.000	0.028	0.049	0.004	0.100	0.044	−0.026	−0.016	0.055	0.141	0.129	0.144	0.235
Religion	0.224	0.333	0.332	0.355	0.387	0.314	0.363	0.366	0.395	0.038	0.028	1.000	0.202	0.088	0.139	0.065	0.024	0.018	0.071	0.242	0.250	0.260	0.386
Relative linked to bullfighting	0.267	0.349	0.349	0.385	0.383	0.303	0.359	0.380	0.386	0.146	0.049	0.202	1.000	0.134	0.278	0.122	0.119	0.022	0.091	0.259	0.265	0.274	0.396
Habitat	0.044	0.095	0.114	0.103	0.104	0.098	0.109	0.107	0.113	0.132	0.004	0.088	0.134	1.000	0.204	0.084	0.090	0.024	0.012	0.079	0.078	0.063	0.122
Region of residence	0.167	0.310	0.291	0.316	0.339	0.285	0.313	0.314	0.341	0.080	0.100	0.139	0.278	0.204	1.000	0.064	0.076	0.033	0.050	0.190	0.198	0.206	0.358
Education	0.060	0.067	0.128	0.097	0.091	0.052	0.100	0.116	0.095	0.107	0.044	0.065	0.122	0.084	0.064	1.000	0.062	−0.095	0.061	0.101	0.100	0.090	0.105
Occupation	0.079	0.109	0.105	0.104	0.106	0.122	0.124	0.108	0.108	0.521	−0.026	0.024	0.119	0.090	0.076	0.062	1.000	0.058	0.146	0.128	0.136	0.136	0.122
Professional field is veterinary medicine	0.017	0.058	0.033	0.021	0.020	0.030	0.024	0.011	0.026	0.039	−0.016	0.018	0.022	0.024	0.033	−0.095	0.058	1.000	0.044	0.020	0.010	0.010	0.018
Net monthly income	0.061	0.103	0.096	0.091	0.115	0.074	0.113	0.089	0.113	0.096	0.055	0.071	0.091	0.012	0.050	0.061	0.146	0.044	1.000	0.066	0.070	0.080	0.111
Suffering capacity of bulls (with respect to dolphins)	0.485	0.450	0.455	0.473	0.506	0.425	0.456	0.472	0.508	0.146	0.141	0.242	0.259	0.079	0.190	0.101	0.128	0.020	0.066	1.000	0.857	0.761	0.507
Suffering capacity of bulls (with respect to a dogs)	0.488	0.471	0.472	0.493	0.522	0.447	0.484	0.495	0.529	0.163	0.129	0.250	0.265	0.078	0.198	0.100	0.136	0.010	0.070	0.857	1.000	0.805	0.528
Suffering capacity of bulls (with respect to humans)	0.493	0.495	0.485	0.515	0.545	0.462	0.504	0.517	0.554	0.156	0.144	0.260	0.274	0.063	0.206	0.090	0.136	0.010	0.080	0.761	0.805	1.000	0.547
Attendance to bullfighting shows	0.518	0.784	0.768	0.813	0.888	0.736	0.806	0.790	0.912	0.140	0.235	0.386	0.396	0.122	0.358	0.105	0.122	0.018	0.111	0.507	0.528	0.547	1.000
